# Supplementary material for: Açaí (Euterpe oleracea Mart.) Seed Extracts from Different Varieties: A Source of Proanthocyanidins and Eco-Friendly Corrosion Inhibition Activity
Source: Molecules. 2021 Jun 5;26(11):3433. doi: 10.3390/molecules26113433 (PMC8201347; doi:10.3390/molecules26113433)
Supplement: Supplementary file 1 [file molecules-26-03433-s001.zip › molecules-1225036-supplementary.pdf]

Article

Açaí (*Euterpe oleracea* Mart.) Seed Extracts from Different Varieties: A Source of Proanthocyanidins and Eco-Friendly Corrosion Inhibition Activity

Gabriel Rocha Martins [1], Douglas Guedes [2], Urbano Luiz Marques de Paula [1], Maria do Socorro Padilha de Oliveira [3], Marcia Teresa Soares Lutterbach [4], Leila Yone Reznik [2],
Eliana Flávia Camporese Sérvulo [2], Celuta Sales Alviano [5], Antonio Jorge Ribeiro da Silva [1] and Daniela Sales Alviano [5,*]

1 Instituto de Pesquisas de Produtos Naturais, Bloco H, Centro de Ciências da Saúde, Universidade Federal do Rio de Janeiro. Av. Carlos Chagas Filho, 373, Cidade Universitária, Rio de Janeiro, 21941-902, Brazil; gabrielrmartins@gmail.com (G.R.M.); urbanomarques@outlook.com (U.L.M.d.P.); ajorge@ippn.ufrj.br (A.J.R.d.S.)
2 Escola de Química, Bloco E, Centro de Tecnologia, Universidade Federal do Rio de Janeiro. Av. Athos da Silveira Ramos, 149, Cidade Universitária, Rio de Janeiro 21941-909, Brazil; douglas.guedes.ferreira@gmail.com (D.G.); lreznik@eq.ufrj.br (L.Y.R.); eliana@eq.ufrj.br (E.F.C.S.);
3 Embrapa Amazônia Oriental–Trav. Dr. Enéas Pinheiro, s/n.–Belém, Pará, 66095-100, Brazil; socorro-padilha.oliveira@embrapa.br
4 Instituto Nacional de Tecnologia, Divisão de Degradação e Corrosão. Av. Venezuela, 82, Rio de Janeiro, 20081-312, Brazil; marcia.lutterbach@int.gov.br
5 Instituto de Microbiologia Paulo de Góes, Bloco I, Centro de Ciências da Saúde, Universidade Federal do Rio de Janeiro. Av. Carlos Chagas Filho, 373, Cidade Universitária, Rio de Janeiro, 21941-902, Brazil; alviano@micro.ufrj.br
* Correspondence: danialviano@micro.ufrj.br; Tel.: +5521-3938-6711

SUPPLEMENTARY MATERIAL

Figure S1 – HILIC-HPLC-FLD chromatograms of PA, WA and BRS crude extracts. Peaks are identified by their degree of polymerization (DP).
Figure S2 – HPLC-DAD chromatogram of PA extract. Extracted ion chromatograms corresponding to B-type procyanidins oligomers (monomer, dimer, trimer, tetramer, pentamer, hexamer, heptamer and octamer, respectively).
Figure S3 – HPLC-DAD chromatogram of WA extract. Extracted ion chromatograms corresponding to B-type procyanidins oligomers (monomer, dimer, trimer, tetramer, pentamer, hexamer, heptamer and octamer, respectively).
Figure S4 – HPLC-DAD chromatogram of BRS extract. Extracted ion chromatograms corresponding to B-type procyanidins oligomers (monomer, dimer, trimer, tetramer, pentamer, hexamer, heptamer and octamer, respectively).
Figure S5 – Chromatograms of PA, WA and BRS phloroglucinolysis aqueous fractions.
Figure S6 – MALDI-TOF $[M+Na]^+$ mass spectra from PA, WA and BRS aqueous fractions.
Figure S7 – Effect of PA on the metallic surface after 24 hours immersion in neutral pH corrosive solution without PA and 1.0 $g.L^{-1}$ of PA. A – Metallic surface after 24 hours immersion in neutral pH corrosive solution; B – Black protective film formation after 24 hours immersion in neutral pH corrosive solution with 1.0 $g.L^{-1}$ of PA; and C – metallic surface after black film removal.
Figure S8 – Nyquist plot for carbon steel AISI 1020 in a neutral pH corrosive solution for purple a ça í(PA) crude extract in 1.0 $g.L^{-1}$. The control represents the absence of any inhibitor.
Figure S9 – Bode plot for carbon steel AISI 1020 in a neutral pH corrosive solution purple a ça í(PA) crude extract in 1.0 $g.L^{-1}$. The control represents the absence of any inhibitor. Relation between log frequency (Hz) and phase angle (°) and log impedance modules ($\Omega \cdot cm^{-2}$).

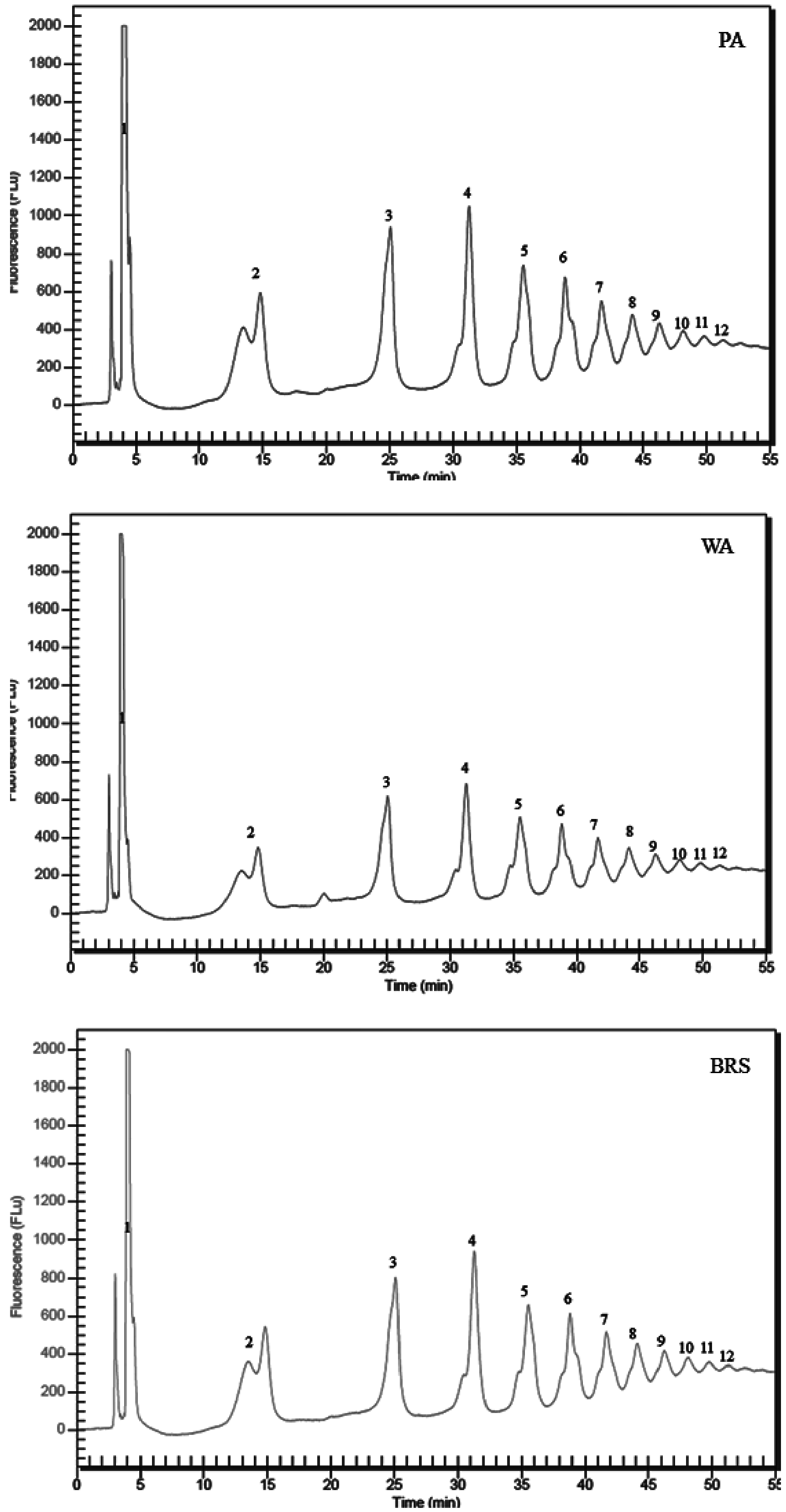

Figure S1 – HILIC-HPLC-FLD chromatograms of PA, WA and BRS crude extracts. Peaks are identified by their degree of polymerization (DP).

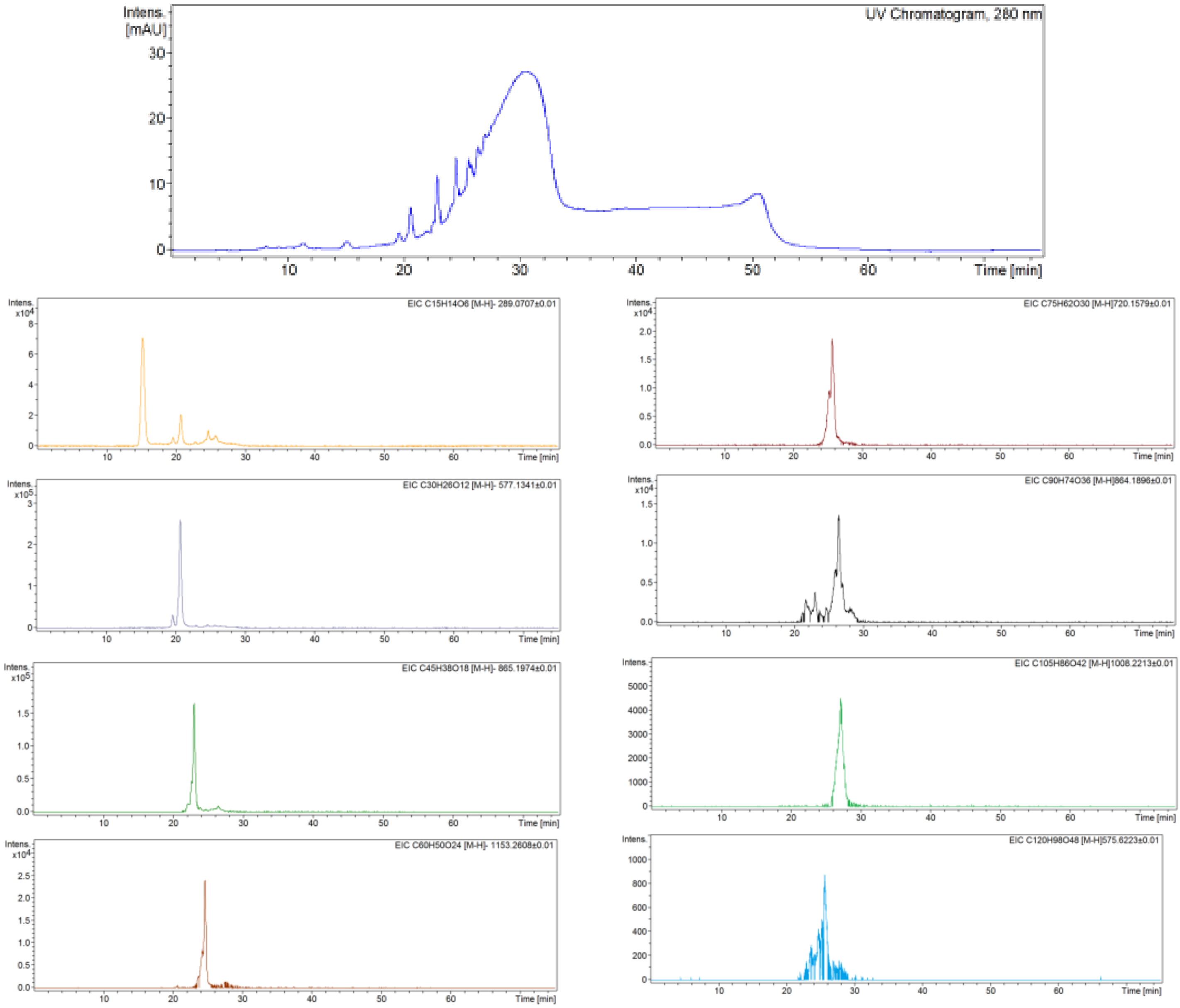

Figure S2 – HPLC-DAD chromatogram of PA extract. Extracted ion chromatograms corresponding to B-type procyanidins oligomers (monomer, dimer, trimer, tetramer, pentamer, hexamer, heptamer and octamer, respectively).

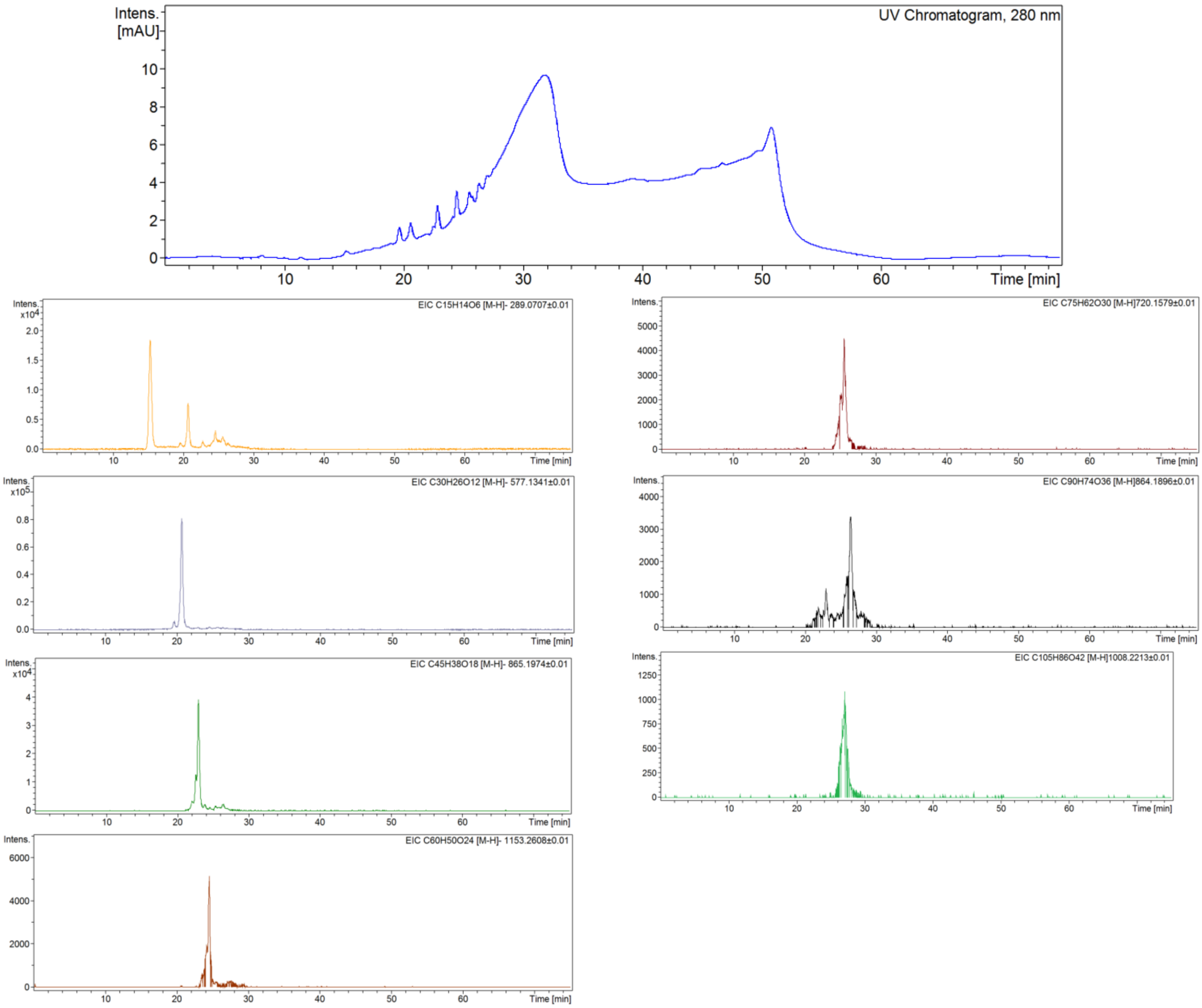

Figure S3 – HPLC-DAD chromatogram of WA extract. Extracted ion chromatograms corresponding to B-type procyanidins oligomers (monomer, dimer, trimer, tetramer, pentamer, hexamer, heptamer and octamer, respectively).

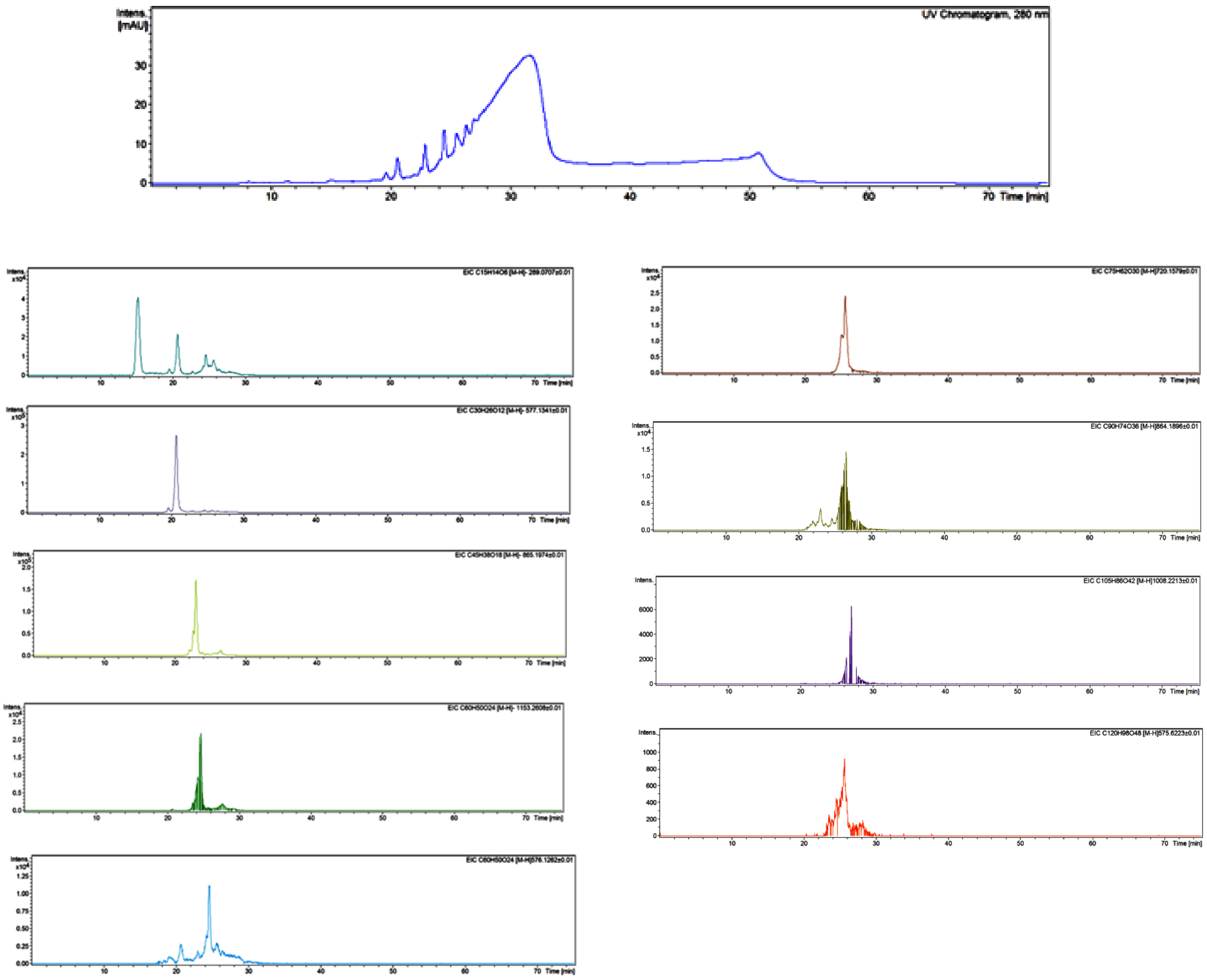

Figure S4 – HPLC-DAD chromatogram of BRS extract. Extracted ion chromatograms corresponding to B-type procyanidins oligomers (monomer, dimer, trimer, tetramer, pentamer, hexamer, heptamer and octamer, respectively).

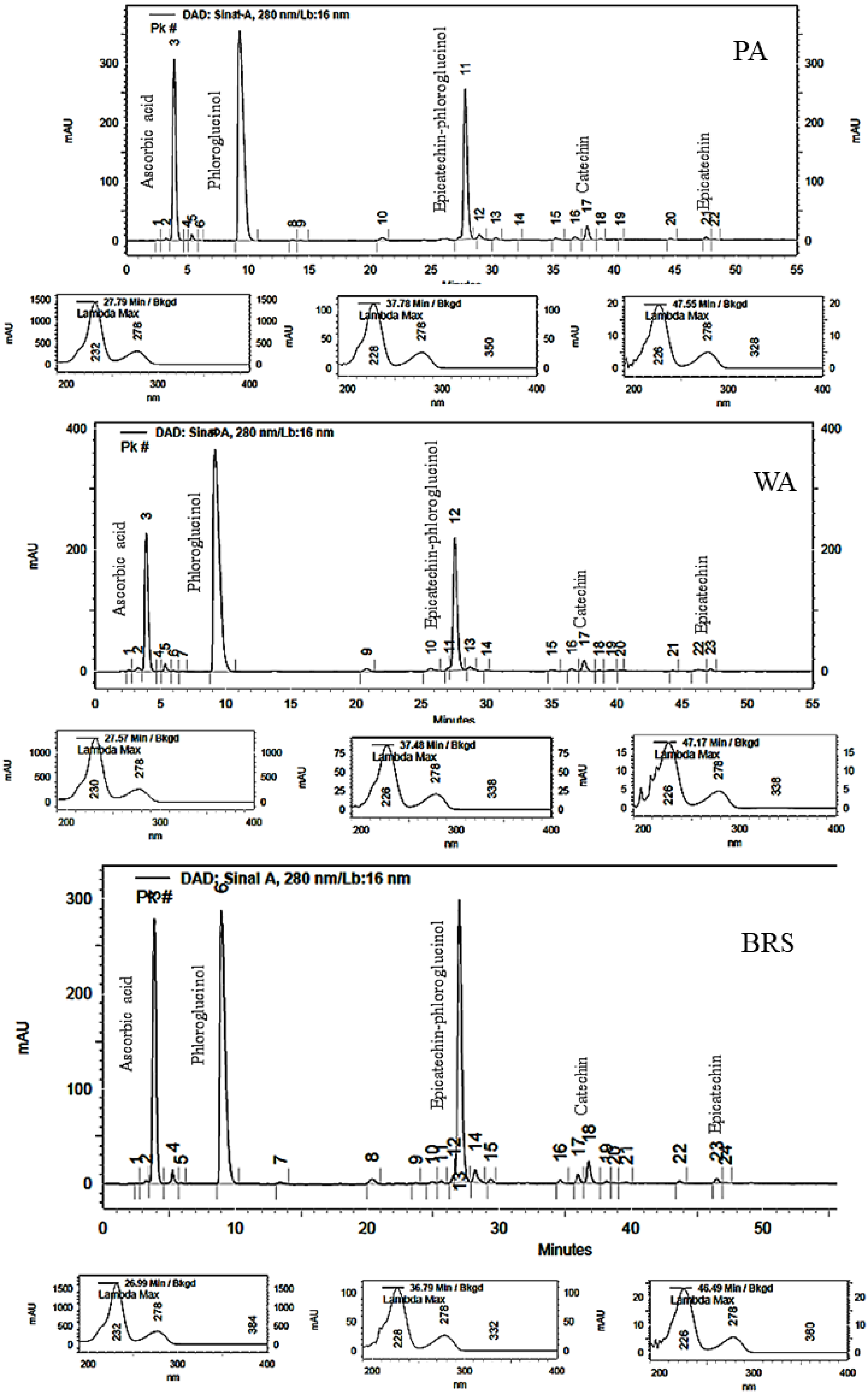

Figure S5 – Chromatograms of PA, WA and BRS phloroglucinolysis aqueous fractions.

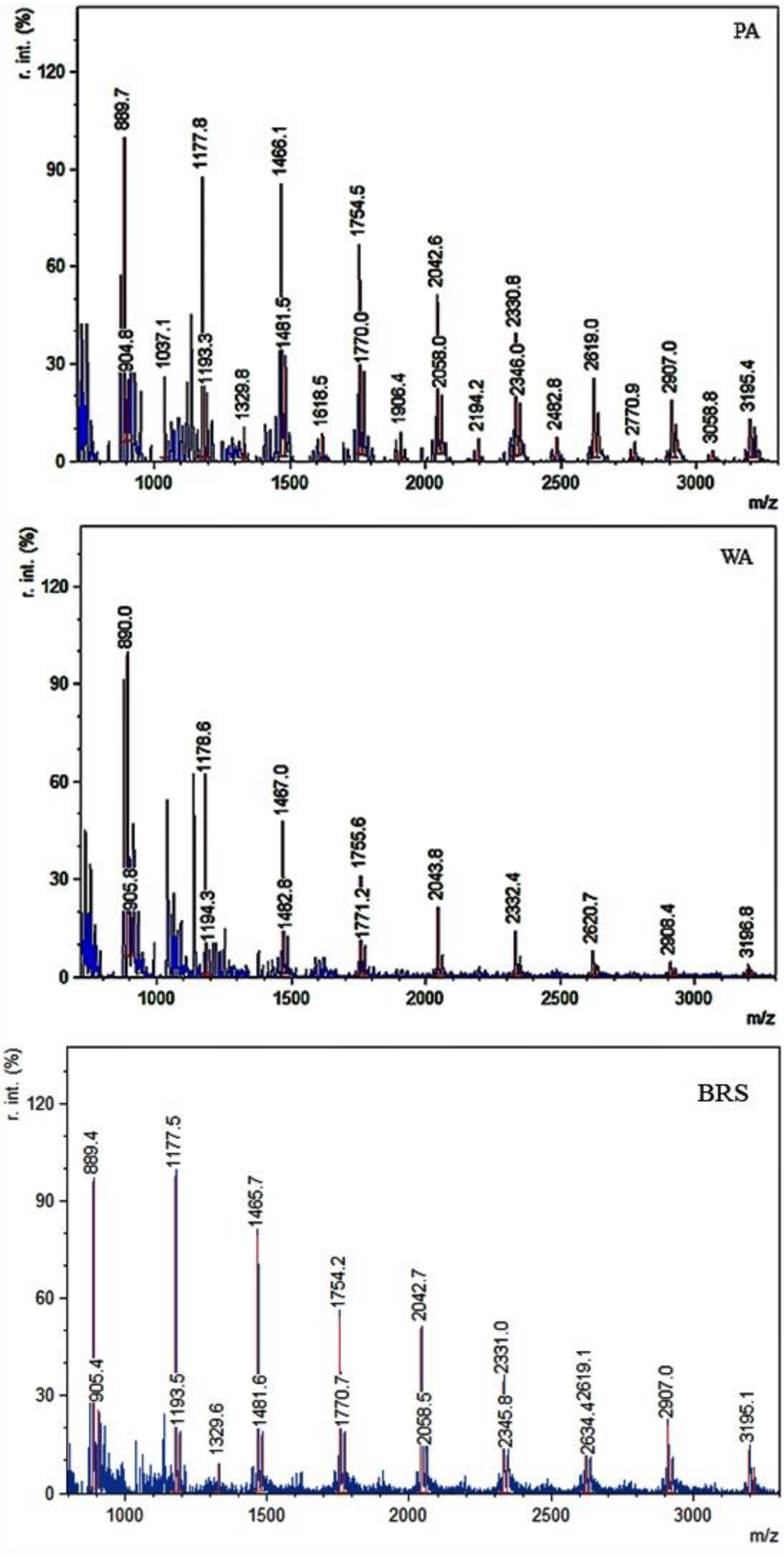

Figure S6 – MALDI-TOF $[M+Na]^+$ mass spectra from PA, WA and BRS aqueous fractions.

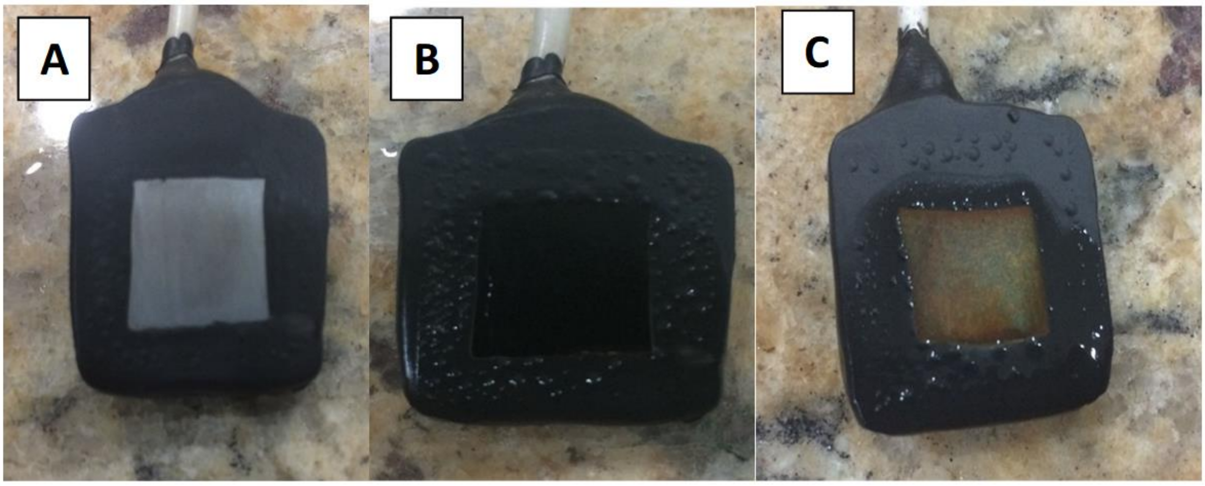

Figure S7 – Effect of PA on the metallic surface after 24 hours immersion in neutral pH corrosive solution without PA and 1.0 $g.L^{-1}$ of PA. A – Metallic surface after 24 hours immersion in neutral pH corrosive solution; B – Black protective film formation after 24 hours immersion in neutral pH corrosive solution with 1.0 $g.L^{-1}$ of PA; and C – metallic surface after black film removal.

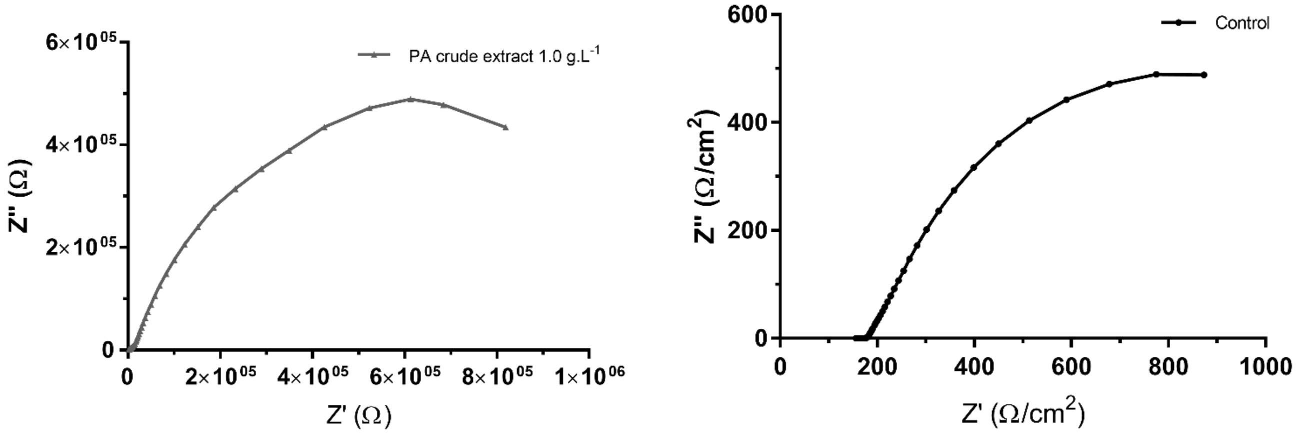

Figure S8 – Nyquist plot for carbon steel AISI 1020 in a neutral pH corrosive solution for purple a ça í(PA) crude extract in 1.0 $g.L^{-1}$. The control represents the absence of any inhibitor.

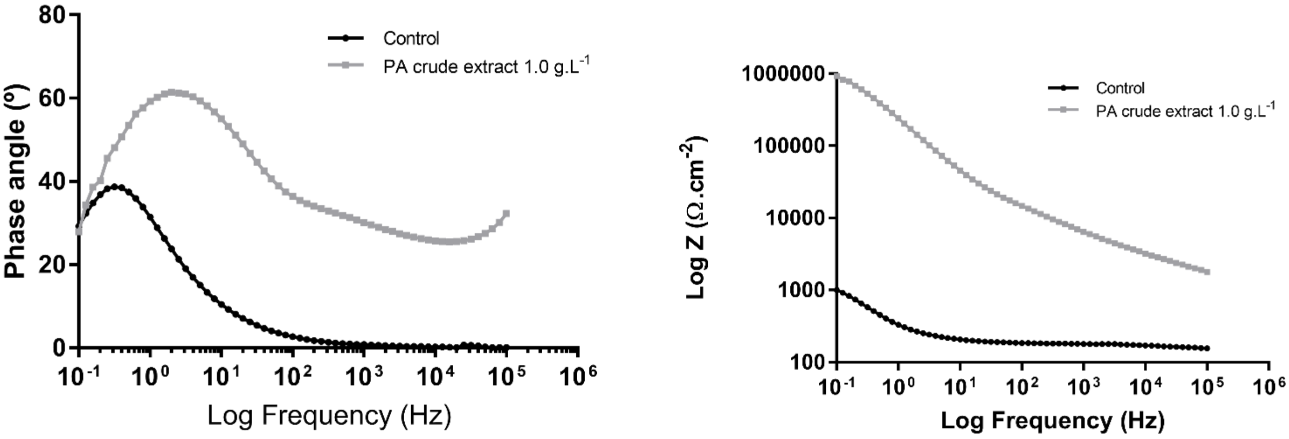

Figure S9 – Bode plot for carbon steel AISI 1020 in a neutral pH corrosive solution purple a ça í(PA) crude extract in 1.0 g.L^{-1}. The control represents the absence of any inhibitor. Relation between log frequency (Hz) and phase angle (°) and log impedance modules ($\Omega\cdot cm^{-2}$).
